# Facial Reconstruction Using Sternocleidomastoid (SCM) Flap: A Review of the Literature

**DOI:** 10.7759/cureus.34575

**Published:** 2023-02-02

**Authors:** Bader Fatani, Abdulaziz A Alabood, Nourah M Alkhayatt, Hadeel H Alzahrani, Afraa Al-Safadi

**Affiliations:** 1 Dentistry, College of Dentistry, King Saud University, Riyadh, SAU; 2 College of Dentistry, King Saud University, Riyadh, SAU; 3 College of Dentistry, Princess Nourah Bint Abdulrahman University, Riyadh, SAU; 4 Surgery and Pharmacy, King Khaled University Hospital, King Saud University Medical City, Riyadh, SAU

**Keywords:** complication, surgery, head and neck, facial reconstruction, sternocleidomastoid flap

## Abstract

The sternocleidomastoid (SCM) flap has been used for a long time in protective coverage of major vessels, reconstruction of intraoral pharyngeal, closure of pharyngo-cutaneous fistulas, and augmentation of soft tissue defects in the oral and maxillofacial region. However, this flap is not yet commonly used due to doubtful blood supply to the flap. This flap offers favorable esthetic results, combined flap, rich vascularization, and the possibility of shifting the two heads of the muscle. Thus, this flap has been used broadly in the maxillofacial region to reconstruct the defects of the post-parotidectomy, mandible, pharynx, and floor of the mouth defects. Previous studies discussed the use of a SCM flap following parotidectomy. However, few studies explained the use of SCMs in facial reconstruction. This study aims to review published articles discussing the use of SCMs for facial reconstruction.

## Introduction and background

Head and neck defects are considered a challenge among surgeons due to the need for reconstruction of the defect according to the complexity of organs involved in the head and region. In addition to concerns related to the capacity to restore function and esthetics [1-2]. The sternocleidomastoid (SCM) flap was first demonstrated in 1882 as the treatment of choice for facial paralysis [3-4]. The SCM flap has the ability to treat various defects including the cheek, floor of the mouth, lower jaw, masticatory function, oropharyngeal, pharyngocutanous, and cervical esophageal fistula [1-2]. The main role of the SCM flap is to restore the missing soft tissues such as in parotidectomy and Frey’s syndrome [5-7]. Multiple approaches for SCM flap were suggested to reconstruct the head and neck defects such as the muscle flap, myocutaneous flap, myoperiosteal flap, and myosseus or osteomuscular flap [8]. This flap can be categorized into two main bases which include the superior or inferior base [1, 8-9]. It is a convenient and reliable flap that provides an adequate substitute to the free flaps, especially in the reconstruction of oral cavity and neck defects after cancer ablation. This flap offers favorable esthetic results, possible use as a combined flap, rich vascularization, and the possibility of shifting the two heads of the muscle [3]. Previous studies discussed the use of a SCM flap following parotidectomy. However, few studies discussed the use of SCMs in facial reconstruction. This study aims to review published articles discussing the use of SCMs for facial reconstruction.

## Review

Methods

This article involved a review of published studies discussing the use of SCM flaps in facial reconstruction. Databases such as PubMed and Google Scholar were used to gather the most relevant studies. A search set was applied to combine a range of keywords: SCM flaps, facial reconstruction, and complication. By using this process, all the papers discussing the use of SCM flaps in facial reconstruction were obtained. In the inclusion criteria, we included all the relevant studies discussing SCM flaps and their complications. The studies that had poor methodological quality and insufficient data were excluded. The initial screening revealed 181 papers. After applying our inclusion criteria, the most relevant articles were selected and used in our current review. This study was conducted by reviewing 32 studies related to the use of SCM flaps in facial reconstruction.

Facial reconstruction

Facial reconstruction has been a debatable topic for surgeons and the public for a long time. Facial reconstruction's main goal is to restore function, while also ensuring a better esthetic outcome. Currently, facial reconstruction has progressed due to detailed anatomic knowledge, which permits tissue transmission from the local or distant region. The chief concern in esthetic facial reconstruction is the analysis of detailed defects. This also involves all reconstructive surgery that requires restoration of function. In defect analysis, a list of the problems that include missing tissues and functional impairment needs to be considered. When all the requirements have been acknowledged, a treatment plan can be prepared. Reconstruction of function must be the cornerstone of facial reconstruction and the starting point for every treatment plan for every surgeon, then the reconstruction of esthetics can be considered [10]. For many years, the free flaps were the principal approach for facial repairs; this includes the conventional anterolateral thigh perforator flap and the radial forearm free flap. With the progress of medical expertise, surgeons have studied the esthetic function of the flap. Better cosmesis and less donor site morbidity have become the main benefits of reconstruction [11]. 

Anatomy of the sternocleidomastoid muscle

The SCM is a long strap-shaped muscle that originates from the medial third of the clavicle and the superficial part of the manubrium of the sternum. This muscle passes posteriorly and superiorly in the neck region and is inserted into the lateral third of the superior nuchal line and the mastoid process [12]. It starts with two heads, the first one is medially from the sternum and the second is laterally from the clavicle. The clavicular head has an aponeurotic and fleshy fiber that arises from the superior-anterior surface of the clavicle. The medial head is composed of a tendinous structure ventrally and arises from the cranial part of the manubrium sterni [13]. The muscle is overlayed by the platysma muscle and is covered by the superficial cervical fascia. The SCM is superficially passed by a sensory branch from the greater auricular, transversus colli, and supraclavicular nerves. In addition for the external jugular vein that is crossed vertically in the neck region. The muscle superimposes the lower third of the carotid sheath and the infrahyoid muscles. The SCM muscle is supplied by the spinal accessory nerve, this nerve passes between the internal carotid artery and the internal jugular vein external to the carotid sheath and enters the deep surface at the carotid bifurcation level [12]. The SCM blood supply can be categorized into the upper third, middle third, and lower third. The upper third originates from branches of the occipital artery. The middle third arises from a branch of the external carotid artery and the superior thyroid artery. The lower third originates from a branch arising from the suprascapular artery (transverse cervical artery) [3, 5, 14-16]. The occipital artery branches are the largest and play the main role in supplying the superiorly based SCM flap [3]. The sternal head of the SCM muscle flap can be planned with greater flexibility compared with the full SCM muscle flap. The sternal head can also offer sufficient tissue mass for the reconstruction of the desired defect, especially in the maxillofacial region [1].

Sternocleidomastoid flap 

The SCM flap was first demonstrated by Jianu in 1882 as the treatment of choice for facial paralysis, nowadays, it can be used for various purposes [2-4, 17-18]. Later on, the use of a SCM flap has been suggested for several treatment options including the reconstruction of intraoral defects, repair of defects following parotidectomy, and reconstruction of the esophagopharyngeal fistula or trachea [3]. The SCM flap has been used for a long time in protective coverage of major vessels, reconstruction of intraoral pharyngeal, closure of pharyngo-cutaneous fistulas, and augmentation of soft tissue defects in the oral and maxillofacial region. However, this flap is not yet commonly used due to doubtful blood supply to the flap [3, 17-18]. Due to its segmental vascular supply, the SCM flap can be used as a superiorly or inferiorly based flap [5, 19]. The skin paddle of the SCM flap is situated above the lower portion of the flap and usually undergoes total or partial necrosis due to the far distance from the occipital artery that limits the use of the flap in various surgeries. The SCM flap is a convenient and reliable flap that provides an adequate substitute to the free flaps, especially in the reconstruction of oral cavity and neck defects after cancer ablation [3]. This flap offers favorable esthetic results, possible use as a combined flap, rich vascularization, and the possibility of shifting the two heads of the muscle. Thus, this flap has been used broadly in the maxillofacial region to reconstruct the defects of the post-parotidectomy, mandible, pharynx, and floor of the mouth defects [4, 19-20]. Asal et al. investigated the incidence of facial contour deformity and Frey's syndrome in patients who had superficial parotidectomy. The results showed that there are no statistically significant differences between these patients in terms of cosmetic outcomes [21]. Moreover, Gooden et al. suggested that the use of a SCM flap after parotidectomy does not significantly improve facial esthetics and contour and does not affect the incidence of Frey's syndrome [7]. Preservation of the cranial portion of the external jugular vein and the superior thyroid arteriovenous system during the harvesting of the flap can reduce the probability of skin paddle necrosis and result in a positive outcome for the skin paddle [3]. The SCM flap can be used to reduce the deformity contour following parotidectomy. However, it forms a deep deformity in the superior part of the neck at the donor site, particularly in young and lean patients [5-6, 22]. This defect usually develops more clearly when a neck movement on the opposite side is done or if a large flap is elevated [5]. A previous study done by Wang et al. showed that the use of the SCM flap for the treatment of chronic facial palsy has provided a strong muscle contraction and an early recovery [4]. For cheek reconstruction, Khazaeni et al. suggested the use of SCM flaps and reported reasonable esthetic results [2]. Moreover, Agarwal et al. demonstrated that lip augmentation with fascia grafts and SCM muscle showed a long-term improvement of the vermilion and lip projection [23]. Facial paralysis can be repaired in the late stage by transposition of the pedicled SCM flap, this method was reported to be effective in restoring both dynamic and static symmetry of mouth and nose, and for recovery of the oral commissure and facial expression [24]. A study conducted by Feng et al. showed that the SCM flap can be considered during the reconstruction of the posterior and inferior tympanic walls. In addition, the author proposed that this flap method does not affect functional recovery or facial nerve transposition if it was performed carefully [25]. One of the defects that is successfully treated with this flap is the cheek lesions where the patient presents with a full thick cheek lesion with exposed molar teeth and an inflamed, tender, nodular lesion that is diagnosed with infiltration squamous cell carcinoma. The SCM muscle can be used as a combination with fascia graft to enhance the vermilion show and lips projection for cosmetic reasons [23]. Moreover, it is considered one of the approaches for the reconstruction of the parotidectomy either due to large benign or malignant metastasis where the loss of facial soft tissue contour can be compensated by the SCM muscle flap [18]. The SCM muscle flap is one of the techniques that can be used after mandible dissection combined with cheek deficiency and augmentation that requires restoration of the normal appearance where it can be accomplished by a composite section of the SCM flap, cervical skin on the muscle, and the medial segment of the clavicle, except in cases with high-risk of deficiencies in the mandible which will require reconstruction with a bony framework by screw-fixation then followed by a SCM flap for augmentation of the deficient cheek [14]. The SCM flap demonstrates its effectiveness in the preservation of hearing by replacing the posterior and inferior walls of the external auditory canal on the lateral skull base lesion [25].

Treatment planning and requirements of reconstruction

The SCM flap is currently used for head and neck reconstruction, closure of tracheal or pharyngoesophageal fistulas, reconstruction of the oral cavity including the floor of the mouth and tongue, reconstruction of the lower jaw, correction of facial paralysis, large vascular neck protector, and coverage of defects after parotidectomy [6, 8, 26]. Ideally, the tissue used should be reliable, functional, and cosmetically acceptable, in addition, the flap should be of sufficient size with minimal morbidity at the donor site and match the recipient site in color, texture, and thickness [3]. The SCM muscle can be used effectively to eliminate the contour deformity after parotidectomy, but consequently will create a deformity at the donor site, especially in thin young patients. This defect becomes more apparent when a large flap is elevated, or the patient performs neck movements on the opposite side. The supra-based SCM flap is the most commonly used due to its constant and known vascular supply from the occipital and superior thyroid arteries. Inferior-based SCM flaps are reserved for lesions in the lower neck and/or superior mediastinum [5]. The SCM muscle flap can be used either as a cranial or inferior flap due to its segmental vascular supply. This flap is supplied by the occipital artery from above, in the middle by the superior thyroid artery, and the transverse cervical artery inferiorly [2, 5, 17]. Anatomical studies have shown three possible branching patterns for the occipital artery and six branching patterns for the superior thyroid artery. These blood supply fluctuations affect flap rotation and perfusion status [2]. The SCM flap can be used in treating head and neck facial defects, whether in the reconstruction of regional defects, rehabilitation of oral cavity defects, or reanimating the face. It can also aid in shoulder elevation and protect the carotid and innominate arteries [3, 13, 15, 25, 27]. Four types of SCM flaps have been described so far: the muscle flap; the myocutaneous flap, in which the muscle is either advanced together with an island of skin, or a composite flap of muscle, connective tissue, and platysma is harvested and covered with split skin; the myoperiosteal flap, in which the muscle is displaced along with a piece of periosteum taken from the clavicle; and the myosseus, or osteomuscular flap, in which the muscle is displaced along with a piece of the clavicle [19]. When creating the SCM flap, certain elements should be considered. First, accessory nerves supply the muscle fibers, so an incision at the SCM origin should preserve the nerve to protect its function. Second, the flap can be separated into two components or added to an occipital myocutaneous flap to rebuild the inferior and posterior walls of the external auditory canal (EAC). Third, when SCM is combined with the spiral EAC flap, it can help improve EAC as there are several types of EAC flaps, and it has been suggested that they can be used for canaloplasty surgery. It can treat various pathologies, preserving the overlying skin as it has a broad base and a vigorous blood supply. In addition, the facial nerve can be better protected when positioned between the two layers of the flap. Finally, sufficient SCM volume should be used to prevent EAC adhesion. The volume required depends on several factors, including the patient's height and weight and whether there is a deficiency in the tympanic cavity [25].

Surgical approach

**Figure 1:**
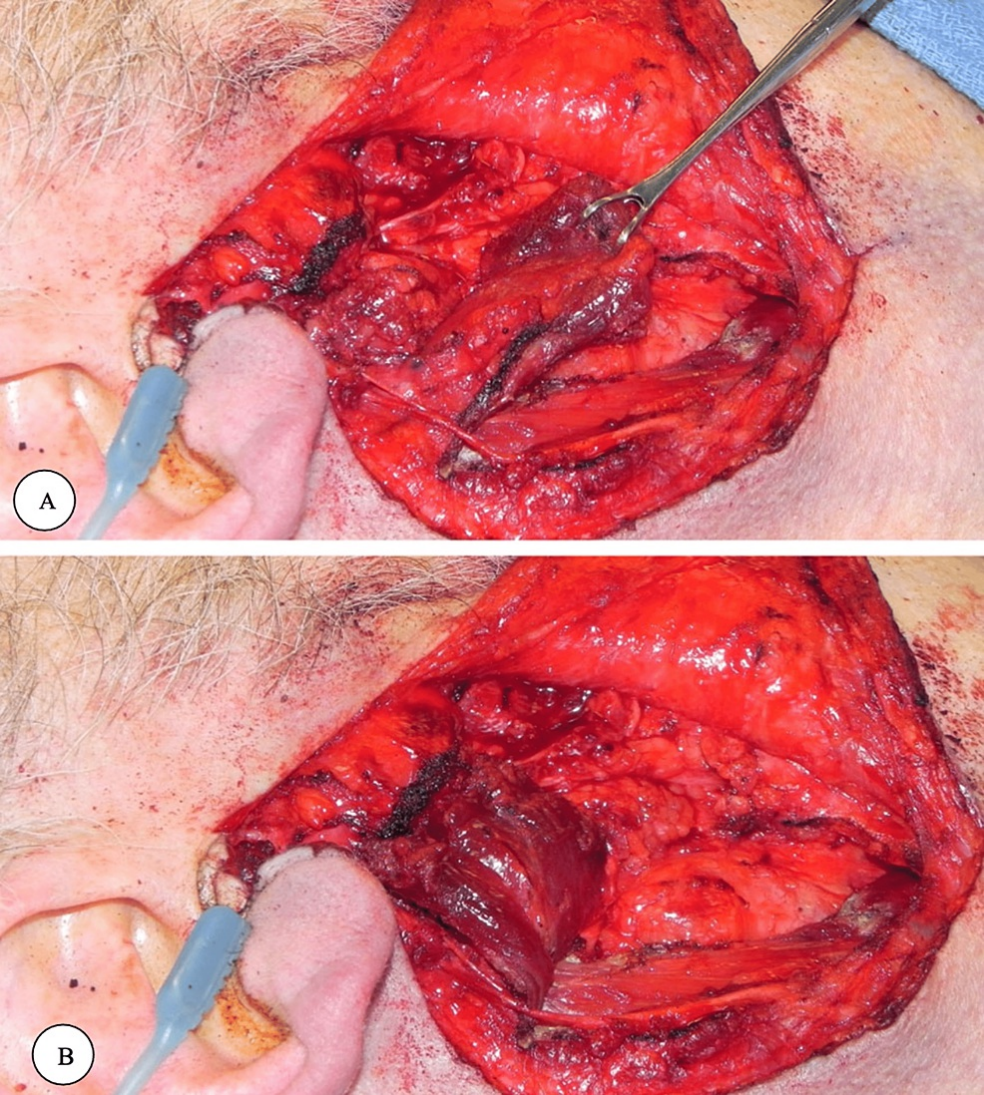
A) Demonstration of superiorly based sternocleidomastoid flap raised and rotated. B) The flap is then sutured to the remaining sub-superficial musculoaponeurotic system in the parotid defect. © The Author(s). 2020 Open Access This article is licensed under a Creative Commons Attribution 4.0 International License, which permits use, sharing, adaptation, distribution and reproduction in any medium or format, as long as you give appropriate credit to the original author(s) and the source, provide a link to the Creative Commons licence, and indicate if changes were made [6].

Flap thickness varies between cases corresponding to the size of the gap and type of surgery (whether it was superficial or total parotidectomy), and the flap length is determined by measuring the defect length before cutting the SCM. Care should be acquired to avoid any injury to the accessory spinal nerve during the flap's cutting and dissection. The flap should be sutured to cover the parotid bed from the zygomatic arch above to the level below the mandible. Then the suction drainage should be placed deep on the flap before closing the wound [8]. The design of the flap follows an imaginary line that runs across the SCM in the fiber direction; a skin paddle is incised over the muscle in a distal position to allow maximum arc of rotation [15, 28]. After selection, the skin paddle is dissected by incising it down to the underlying muscle without cutting it. The flap is then entirely exposed by dissecting the muscle pedicle; At this point, the flap is ready to be transferred to the reconstructive site [15]. This flap must be planned with the inversion method to show if it can easily reach the defect. Suturing the skin's dermis or underlying soft tissue paddle to the muscle helps avoid avulsion during flap manipulation. The skin itself should not be included in this fixation. If necessary, the superior thyroid artery can be ligated in the superior lobe for the best arc of rotation, leaving only the occipital and/or posterior auricle arteries [15]. While most authors recommended the preservation of at least two arteries, in this series, all skin flaps were supplied by only one (the occipital artery). However, in each case, the skin flap was designed just over the muscle and not beyond, according to the original concepts by [15, 17]. Occasionally, the arch of rotation may be restricted by the accessory nerve. Intravenous fluorescein can be used to determine skin paddle viability after lifting, although this is unreliable. The SCM myocutaneous flap generally allows for easy closure of the donor defect [15]. To expose the entire length of the SCM, the main line incision started from the helical crus to the earlobe, rotated around the mandibular angle to the mastoid process, and then extended down the posterior border of the SCM to the sternoclavicular joint. An auxiliary line should be drawn along the nasolabial fold to the inferior oral commissure to expose the orbicularis oris. After flap reflection by elevating the platysma muscle, essential structures, including the external jugular vein, great auricular, and transverse cervical nerves, should be carefully identified. Anterograde dissection is performed to identify and preserve the facial nerve branches [4-5]. Depending on the tumor extent, a superficial or complete parotidectomy is performed [5]. Marx et al. were the first to show that maintaining the thyroid artery intact can considerably improve the SCM flap's survival rate, with just 2 of 16 reported cases resulting in skin necrosis. The author proposed that one artery could provide the skin paddle with enough blood while maintaining as many venous perforators as feasible and maintaining the cranial section of the external jugular vein to offer an additional layer of security [3]. Ibrahim et al. assessed the effect of SCM flap augmentation of the pharyngeal closure after total laryngectomy on the pharyngocutaneous fistula. The results showed that the use of an SCM flap did not reduce the pharyngocutaneous fistula incidence after total laryngectomy [29]. Several recent studies suggested that before rotating the SCM flap, the skin should be sutured to the muscle to protect the skin's small perforators from being lost. It is possible that the preserved SCM branch of the superior thyroid artery contributed to the lower failure rates. It could be possible to guarantee that the entire muscle obtains blood flow by maintaining both the superior thyroid artery and occipital artery branches [30]. The tracheoesophageal fistula can also be reconstructed with the SCM flap rotation flap. In case the tracheoesophageal fistula is suitable in size, a reconstructive plan with an SCM is simple and effective to close the persistent fistula [31]. The occipital myocutaneous flap can be rotated and joined with the SCM flap for mastoid reconstruction in case the SCM flap was insufficiently sizable. Feng et al. discussed the main factors for designing the SCM flap [25]. First, the muscle fibers controlled by the accessory nerve were kept when the SCM was severed at its origin, preserving its function. Second, to repair the inferior and posterior walls of the EAC, the flap was divided into two parts or joined with an occipital myocutaneous flap. The flap is then secured with a silk suture. Third, the SCM flap may aid in the formation of a better EAC when paired with the spiral EAC flap. In order to provide better protection, the facial nerve can be positioned between the flap's two layers. Finally, it is important to employ a suitable amount of SCM since in one of the cases reported in this study, an excessive amount of muscle volume caused EAC adhesion [25]. Demonstration of the superiorly based SCM flap in Figure 1.

Complications

The most common postoperative complications that affects an individual's quality of life can be divided into both cosmetic and functional complications. The most common cosmetic defect associated with SCM is in the donor area due to its association with vast tissue loss [3]. A deformity in the resected area was perceived in all patients and was observed in two patients according to a study by Wang et al. [4]. A scar was also noticeable in all cases, but a severe hypertrophic scar was not observed at the follow-up stage [4]. As for the functional complications, they most commonly include facial nerve paralysis, Frey's syndrome, first bite syndrome, and loss of auditory sensation [3, 6, 22, 32]. 

The standard treatment for benign parotid masses has been superficial parotidectomy, which allows for complete tumor removal while reducing the possibility of recurrence, however, these treatments come with consequences [6]. The parotid gland contains the facial nerve, which is an important nerve. The seventh cranial nerve involves the sensory, motor, and parasympathetic nerve fibers that innervate several regions of the head and neck. Facial nerve paralysis, whether temporary or permanent, is the most disturbing complication of parotid surgery [6, 22]. Frey’s syndrome is a postoperative condition characterized by gustatory sweating and flushing after salivary gland surgery, neck dissection, facelift procedures, and trauma [8]. According to a study by Nofal and Mohamed, when a starch iodine test is performed four days after parotidectomy, the prevalence rate of Frey’s syndrome was 94%, but only 12%-54% in symptomatic patients [8]. It is thought to be caused by abnormal regeneration of the affected parasympathetic fibers. Since the nerve fibers are responsible for innervating the sweat glands of the overlying skin, it will result in gustatory sweating within two weeks to two years [8]. 

**Figure 2:**
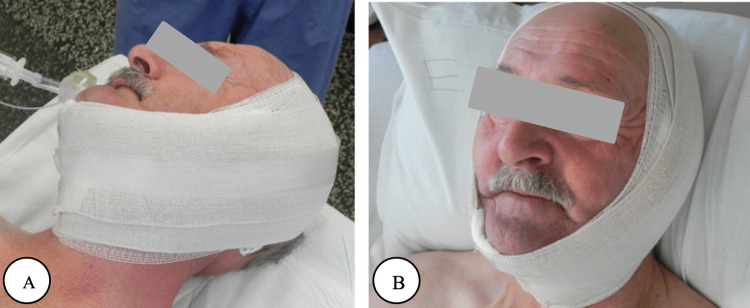
Postoperative placement of dressing. A) Side view. B) Anterior view. © The Author(s). 2020 Open Access This article is licensed under a Creative Commons Attribution 4.0 International License, which permits use, sharing, adaptation, distribution and reproduction in any medium or format, as long as you give appropriate credit to the original author(s) and the source, provide a link to the Creative Commons licence, and indicate if changes were made [6].

The SCM flap has been linked to some complications. As previously discussed with the various approaches, the skin paddle in the conventional superiorly based SCM flap typically undergoes partial or total necrosis because of its far location from the occipital artery, thus limiting its clinical utilization [3]. Infection is another complication caused primarily by the parotidectomy rather than the flap and was reported in previous studies that were successfully treated with antibiotics [15, 18]. Another consequence is the development of sialocele. According to Melong et al. study, two patients developed a postoperative sialocele in the first week following the surgery [6]. The sialocele is drained in the office, and the patients were treated with antibiotics and pressure dressings. They were handled with conservative treatment, without further assistance, and with no long-term effects [6, 18]. Among the cases, there were no deaths and no skin necrosis unless other complicating factors were present, such as infection or a fistula [15]. Postoperative placement of dressing is demonstrated in Figure 2.

## Conclusions

The SCM flap has proven its convenient and reliable properties that offer favorable esthetic results and rich vascularization. In addition to its various uses in the maxillofacial region to reconstruct various defects such as post-parotidectomy and floor of the mouth defects. However, a few considerations must be carefully understood by the practicing surgeon. This flap can form a deep deformity in the superior part of the neck at the donor site, particularly in young patients. In addition, care should be taken by the practicing surgeon to avoid any injury to the accessory spinal nerve during the flap's dissection. Current literature suggests maintaining at least two arteries for the SCM flap while retaining the thyroid artery intact to improve the flap's survival rate. The skin should also be sutured to the muscle to protect the skin's small perforators from being lost. Moreover, surgeons should be aware of the common complications associated with this flap as well as the appropriate management of these complications which include facial nerve paralysis, Frey’s syndrome, first bite syndrome, and loss of auditory sensation.
